# Durum wheat (*Triticum turgidum* L. var. *durum*) root system response to drought and salt stresses and genetic characterization for root-related traits

**DOI:** 10.3389/fpls.2024.1362917

**Published:** 2024-03-22

**Authors:** Luca Bonfiglioli, Ieva Urbanavičiūtė, Mario A. Pagnotta

**Affiliations:** Department of Agricultural and Forest Sciences, Tuscia University, Viterbo, Italy

**Keywords:** durum wheat, abiotic stress, high throughput root phenotyping, SSR molecular markers, genetic diversity

## Abstract

Abiotic stresses such as drought and salt are significant threats to crop productivity. The root system adaptation and tolerance to abiotic stresses are regulated by many biochemical reactions, which create a complex and multigenic response. The present study aims to evaluate the diversity of root responses to cyclic abiotic stress in three modern durum wheat varieties and one hydric stress-tolerant landrace in a pot experiment from seedling to more advanced plant development stages. The genotypes responded to abiotic stress during the whole experiment very differently, and at the end of the experiment, nine out of the 13 traits for the landrace J. Khetifa were significantly higher than other genotypes. Moreover, single sequence repeat (SSR) genetic analysis revealed high polymorphism among the genotypes screened and interesting private alleles associated with root system architecture traits. We propose that the markers used in this study could be a resource as material for durum wheat breeding programs based on marker-assisted selection to increase the vegetal material with high drought and salt stress tolerance and to identify candidates with strong early vigor and efficient root systems. This study provides appropriate genetic materials for marker-assisted breeding programs as well as a basic study for the genetic diversity of root traits of durum wheat crops.

## 1 Introduction

Drought and salt are the most harmful abiotic stresses for crop productivity all over the world (Mondini and Pagnotta, 2015; Urbanavičiūtė et al., 2021). Particularly in the Mediterranean region, water shortage and salty water irrigation represent critical issues affecting crop yields, specifically for durum wheat, *Triticum turgidum* L. subsp. *durum* (Desf.), one of the most cultivated crops in the region (Carvalho et al., 2014; Rezzouk et al., 2022). Although both stresses have negative impacts on plant growth and development, each one affects plants differently (Oliveira et al., 2013; Golldack et al., 2014). The consequence of water deficit, in addition to osmotic stress, is that plants also suffer the toxic effects of accumulated Na^+^ and Cl^−^ ions in the presence of salt (Munns and Gilliham, 2015; Liang et al., 2018). Leaves represent the final sink and the most vulnerable part of the plant exposed to the atmosphere where salt accumulates, and this decreases the availability of nutrients. Understanding the mechanisms by which these genotypes maintain relatively low cellular Na^+^/K^+^ levels is crucial for salt tolerance (Soni et al., 2022). Overall, complex factors are involved in response to stresses, which are mainly determined by the environment and the genetic capacity of a plant to recognize and respond to adverse conditions. Moreover, the impacts of abiotic stresses on plants depend on their timing, severity, and dose. It was reported that a major loss of production in wheat occurs when drought events affect the plants in early stages, such as the tillering and stem elongation stage (Blum et al., 1990; Saeidi et al., 2015). It was also demonstrated that salinity applied before terminal spikelet differentiation (TSD) caused a reduction in the number of spikelets per spike and number of tillers per plant, whereas salinity imposed after TSD significantly reduced only the kernels’ number and weight (Francois et al., 1994). Moreover, it was shown that severe drought stress reduced wheat grain quality, while moderate stress improved its quality (Ozturk et al., 2022). Furthermore, grain yield reduction varied among durum wheat genotypes under different salt concentrations (Husain et al., 2003). The harmful effects on crop yield also depend on stress frequency, such as a single severe drought or mild cyclic drought events (Izanloo et al., 2008; Ding et al., 2018).

Plant roots are essential to detect and respond to abiotic stresses. However, the plasticity of their growth and development in response to changing soil conditions is crucial and remains a challenge (Shelden and Munns, 2023). Recently, several studies have highlighted relevant root adaptation strategies as a reaction to abiotic stresses and have shown the root system importance for plant performance (Chen et al., 2018; El Hassouni et al., 2018; Dwivedi et al., 2020; Zou et al., 2021). The root angle, for example, was defined as a principal trait for the selection of drought tolerance genotypes (Wasson et al., 2012; Urbanavičiūtė et al., 2022a). It was found that genotypes with a narrow root angle had longer roots under drought conditions and had better water availability from deeper soil layers (Richard et al., 2015; Chen et al., 2018; El Hassouni et al., 2018). However, it was demonstrated that shallower root growth could enhance rice yields in a salty environment (Kitomi et al., 2020). The impact of abiotic stress on root angle is associated with gravitropism *versus* anti-gravitropism regulatory mechanism. Under drought, roots grow toward water in the direction of the gravity vector, while they direct their growth away from salt against the gravity vector through negative halotropism (Sun et al., 2008). Moreover, the gene responsible for gravitropism regulatory mechanisms and root angle was identified (Fusi et al., 2022). The root response to abiotic stress and consequently the root angle is mainly regulated in root tips, where the auxin distribution determines the reorientation of the root along or against gravity as adaptation strategies to avoid unfavorable environments (Waite et al., 2020; Ober et al., 2021). Although several genes responsible for root angle have been identified (Wang et al., 2018; Furutani and Morita, 2021; Fusi et al., 2022), incorporating them into breeding programs remains challenging.

Both the root system plasticity and tolerance to abiotic stress are multigenic traits regulated by several biochemical reactions. However, plants have several adaptation systems that aid in tolerating or resisting the negative impact of abiotic stresses. Although numerous quantitative trait loci (QTLs) were identified to be involved in abiotic stress tolerance (Colasuonno et al., 2021), breeding for drought and salt stress-affected environments is extremely challenging due to the complexity and the multitude of mechanisms adopted by plants to mitigate the stress effect (Reynolds et al., 2005). The marker-assisted selection with molecular markers such as single sequence repeats (SSRs) is still a solid solution for the study of the root architecture of durum wheat cultivars better adapted to water-limited and salinity conditions (Maccaferri et al., 2016; Alahmad et al., 2019). The identification and the introgression of alleles that contribute to drought and salt stress tolerance is a desirable approach for the improvement of drought-susceptible cultivars (Canè et al., 2014). The SSRs represent an effective tool for allelic identification due to their large level of polymorphism and distribution in the whole genome (Maccaferri et al., 2007; Mondini et al., 2009). Although the analysis of SSRs may determine the principal chromosomal region involved in root architecture features (Soriano and Alvaro, 2019), the genetic selection for drought and salt stresses and the validation of the identified markers and QTLs should be conducted in the target environment, ideally involving the design of ideotypes for a specific growth condition (Negisho et al., 2022).

Furthermore, a good and time-tested breeding strategy for abiotic stress tolerance is the evaluation and exploration of natural genetic diversity including wild genotypes, landraces, and modern varieties (Nazco et al., 2012; Soriano et al., 2016). At the same time, the high-throughput root analysis technologies allow us to see more detailed information on root system plasticity, and they can help determine the target root traits for crop improvement under abiotic stress (Nagel et al., 2012; Richard et al., 2015; Urbanavičiūtė et al., 2022b).

The present study aims i) to evaluate the phenotypic and genotypic diversities in root traits for three modern varieties and one landrace of durum wheat, ii) to assess the responses to multiple abiotic stresses such as drought and salt after several cyclic stress events, and iii) to assess the genetic diversity among the modern varieties and the landraces through the analysis based on SSR markers highly linked to genome regions associated with root traits of interest for drought and salt tolerance breeding programs.

## 2 Materials and methods

### 2.1 Plant material

Four genotypes were used in the study: one hydric stress-tolerant landrace J. Khetifa (Mondini et al., 2015) and three modern varieties, Cham1, Azeghar 2-1 (56) (abbreviated as Azeghar), and Sebatel2 (45) (abbreviated as Sebatel) developed in the Mediterranean Basin.

### 2.2 Experiment condition and design

**Figure 1:**
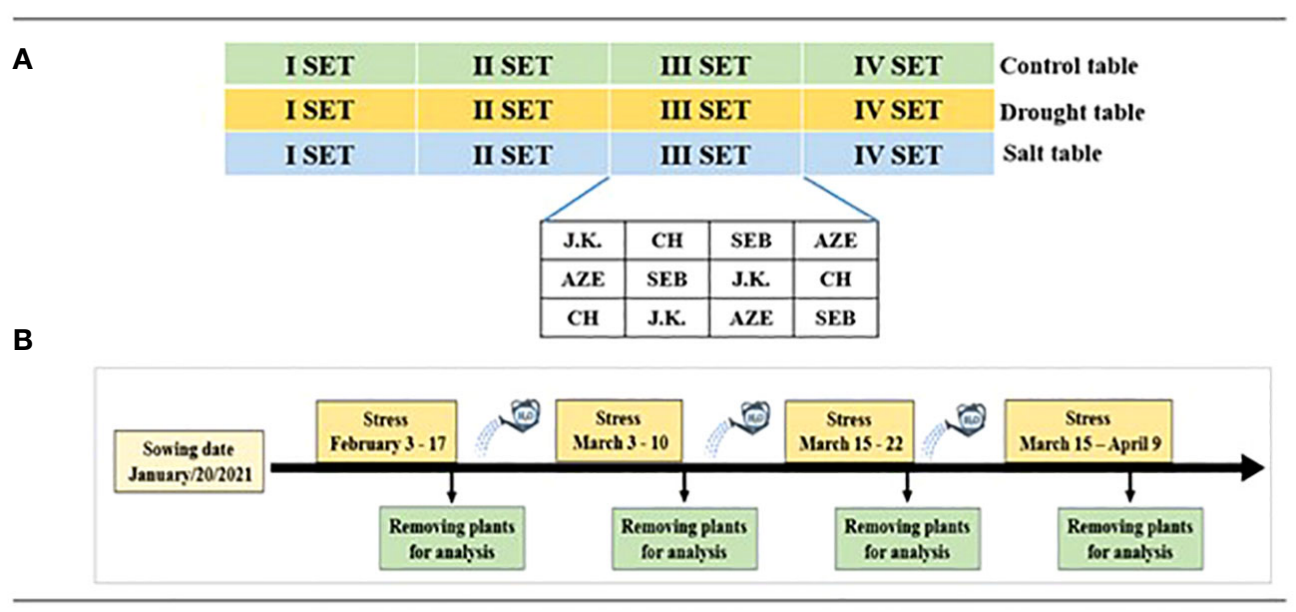
The experimental design. **(A)** Genotype allocation. **(B)** Treatment management.

A total of 144 seeds, 36 per genotype with one seed per pot placed with the embryo facing down, were sown on January 20, 2021, in the greenhouse at Tuscia University experimental farm (Viterbo, Italy; 42°25′29″N 12°04′47″E). Some extra seeds were sown in the same condition to have the possibility of replacing the ungerminated plants. Pots with size of 17-cm diameter by 16-cm height were filled with 2.5 L clean sand. Pots for each treatment (control, drought, and salt) were placed on separate tables with automatic watering. Each table consisted of four sets of 12 plants each disposed in a Randomized Complete Block Design for the four genotypes in three replications (**Figure 1A**). During the experiment, stress (drought and salt) was applied four times. After each treatment event, one set from each table (control, drought, and salt) was removed for analysis, while the remaining treated plants were re-watered to prevent death and treated again (**Figure 1B**). The first drought and salt stress treatments were started when all plants reached the stage where two leaves had unfolded.

The control treatment was irrigated using a dropping pipe three times per week (80 mL/per pot) with a water nutrition solution (**Supplementary Table S1**). Drought treatment was managed by discontinuing watering. The water field capacity (FC; %) of the soil in the pots was monitored by weighing the pots regularly according to Grewal et al. (1990). The salt treatment used the same nutrient solution as the control group, but with the addition of NaCl to reach a concentration of 250 mM. The salt stress level was chosen on the basis of previous experiments to cause a visible effect but not too strong to kill the plants (Mondini et al., 2012; Urbanavičiūtė et al., 2022b).

### 2.3 Plant phenotyping

**Figure 2:**
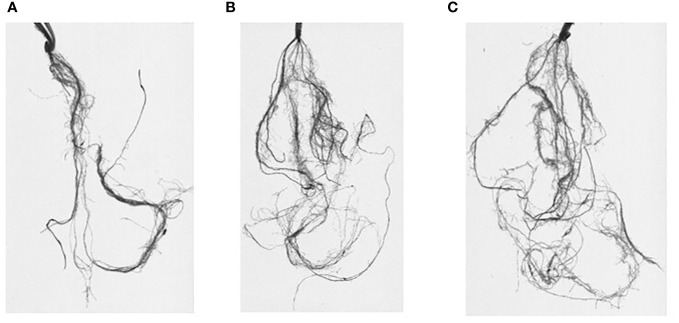
Root systems scanned and analyzed through Win-RHIZO. J. Khetifa roots under control **(A)**, drought **(B)**, and salt stress **(C)** conditions.

Roots were analyzed using Win-RHIZO Pro software v2009 (version 4.0b; Regent Instruments, Montreal, QC, Canada) to obtain root traits such as total root length (RL; cm), surface area (SA; cm^2^), total root volume (RV; cm^3^), number of root tips (TI), forks (FR), and crossings (CR) (**Figure 2**). The root angle (RA; degree on the vertical °) was measured using the software *ImageJ*. After measurements using Win-RHIZO, roots were separated, dried in an oven (at 80°C for 12 h), and weighed for root dry weight (RDW). Plant length (PH) was measured during whole experiments, while the number of leaves (NL) and tillers (NT) was measured only from the second stress event due to the phenological state of plants. After each stress event, plants were carefully removed from pots, and sand was rinsed off using a soft spray watering head.

### 2.4 Plant genotyping

The genetic diversity analysis was conducted using 11 SSRs already identified by Urbanavičiūtė et al. (2023). The markers were already demonstrated as highly associated with chromosomal regions related to root traits (**Supplementary Table S2**). The genomic DNA of each accession was extracted from fresh leaves using the PureLink Plant Total DNA Purification kit (Invitrogen; Thermo Fisher Scientific, Waltham, MA, USA). The PCRs were carried out following the manufacturer’s instruction of GoTaq G2 DNA Polymerase (Promega, Madison, WI, USA) for mixture protocols and cycling conditions. The amplifications were run in SwiftMaxi Thermal Cyclers (Esco Technologies, St. Louis, MO, USA) using different annealing temperatures for each primer (**Supplementary Table S3**). The amplicons were further analyzed through capillary electrophoresis with the device QIAxcel Advanced Instrument (Qiagen, Hilden, Germania) using the QIAxcel DNA High-Resolution Kit, and the amplifications were validated using QIAxcel ScreenGel Software (Qiagen, Hilden, Germany). Each amplicon was named according to its molecular weight; the raw data are reported in **Supplementary Table S4** and the Zenodo repository at the 10.5281/zenodo.10439459.

### 2.5 Statistical analysis

Morphological data were analyzed using R Studio (Version R-4.1.0), and a two-way analysis of variance (ANOVA) was conducted at a significance level of 5% using the aov() function. A one-way ANOVA was used to test the variance component of each trait under each treatment, with genotype as a factor. Fisher’s least significant difference (LSD) test was used to compare means. Regression and correlation analyses between phenotypic and genotypic traits were performed using SPSS (version 14.0 for Windows) statistical software. The genetic analysis was performed using the software GDA (Lewis and Zaykin, 2001) and GenAlEx (Peakall and Smouse, 2012).

## 3 Results

### 3.1 Effects of cyclic drought and salt stresses on shoot traits

After the first stress treatment, the drought and salt stress-tolerant genotype J. Khetifa had a significantly longer seedling length (PH) in both stress conditions. Under the control conditions, the genotypes did not differ from each other (**Table 1**).

**Table 1:** Plant length (PH) (cm) after repeated drought and salt stresses (C, control; D, drought; S, salt).

T	Genotype	1st	2nd	3rd	4th
C	Azeghar	30.3 ± 1a	34.6 ± 3.2b	56.7 ± 3a	76.2 ± 4.4a
C	Cham1	30.1 ± 3.7a	37.5 ± 0.2b	56.7 ± 3.7a	68.2 ± 5.3a
C	JK	37.8 ± 2.8a	42.8 ± 1.1a	48.6 ± 6.2b	55.6 ± 8.9b
C	Sebatel	30 ± 7a	36.4 ± 2.8b	51.1 ± 3.3ab	72.7 ± 5.9a
D	Azeghar	25.2 ± 3.6c	36.7 ± 1.5a	41.3 ± 5.6a	58.4 ± 6.5a
D	Cham1	30 ± 2.7bc	30.5 ± 2.9a	41.7 ± 7a	53.9 ± 2.6ab
D	JK	37.7 ± 3.7a	33.5 ± 3.6a	44.3 ± 3.9a	39.5 ± 1.1c
D	Sebatel	32.3 ± 0.6ab	34.1 ± 5.6a	48.4 ± 1a	51.2 ± 0.8b
S	Azeghar	27.6 ± 1.3bc	40.5 ± 1.6a	46.4 ± 7.9a	54.3 ± 4.2a
S	Cham1	24.4 ± 1.8c	31.3 ± 0.8b	43.2 ± 2.9a	52.8 ± 2.2a
S	JK	38.8 ± 0.3a	33.1 ± 2.6b	38 ± 3.8a	50.4 ± 0.9a
S	Sebatel	30.5 ± 3.1b	34.8 ± 0.8b	47.5 ± 15.1a	48.2 ± 4.5a
	Genotype (G)	***	*	ns	***
	Treatments (T)	ns	**	***	***
	G × T	ns	**	ns	*

Values are means ± standard deviations (n = 3). Means with the same letter in each column, within treatment, are not significantly different between genotypes (p < 0.05) (LSD test).

ns, not significant; LSD, least significant difference.

*, **, and *** indicate significance at p < 0.05, p < 0.01, and p < 0.001 levels, respectively.

Overall, a significant effect of treatments was not detected. However, genotype Cham1 at early stages was more sensitive to salt than to drought stress. After the second stress treatment, significant differences between genotypes, treatments, and their interaction (G × T) were detected in terms of PH (**Table 1**). Both drought and salt stresses had undesirable effects on the PH of Cham1 and J. Khetifa; conversely, the PH of Sebatel was not affected at all. Furthermore, Azeghar PH increases slightly under salt stress compared with the control conditions. After the third stress treatment, PH was affected negatively for all the genotypes. The PH of Sebatel had no significant differences between control and stress conditions, while in J. Khetifa, a major negative effect was detected from salt treatment rather than drought. Cham1 reduced PH under drought and salt conditions, while Azeghar reduced PH only under drought. After the fourth drought and salt stress event, a significant reduction of PH was recorded; the reduction was similar for Azeghar, Cham1, and Sebatel under drought or salt conditions. However, the PH of J. Khetifa was more affected by drought stress than by salt stress.

After two stress events, the number of leaves (NL) significantly increased for Azeghar and J. Khetifa, while Sebatel and Cham1 had no significant differences between control and stress conditions (**Table 2**).

**Table 2:** Number of leaves (NL) and number of tillers after repeated drought and salt stresses (C, control; D, drought; S, salt).

		Number of leaves			Number of tillers		
T	Genotype	2nd	3rd	4th	2nd	3rd	4th
C	Azeghar	5 ± 0b	15.7 ± 2.9c	19.7 ± 3.1b	1 ± 0c	3 ± 0c	3.7 ± 0.6b
C	Cham1	5 ± 0b	23 ± 2b	25 ± 1b	2 ± 0b	4.7 ± 0.6b	4.3 ± 0.6b
C	JK	9.3 ± 0.6a	36.7 ± 1.2a	96.3 ± 6a	3 ± 0a	9 ± 0a	24.3 ± 1.2a
C	Sebatel	5.7 ± 0.6b	17 ± 3.6c	25.7 ± 4.5b	1 ± 0c	3.7 ± 0.6c	4.7 ± 0.6b
D	Azeghar	7 ± 0b	7 ± 0c	14 ± 4.6b	1 ± 0b	1 ± 0c	3 ± 1b
D	Cham1	8 ± 1.7b	8.7 ± 1.5bc	18 ± 3b	1 ± 0b	2.7 ± 1.2b	3.3 ± 0.6b
D	JK	11.7 ± 0.6a	16 ± 1a	36.3 ± 8.5a	3 ± 0a	4.3 ± 0.6a	6 ± 1.7a
D	Sebatel	7.3 ± 2.1b	9.7 ± 0.6b	13.7 ± 1.2b	1.3 ± 0.6b	2.3 ± 0.6b	3 ± 0b
S	Azeghar	6.7 ± 0.6b	6 ± 1b	6.7 ± 0.6b	1 ± 0b	1 ± 0b	1 ± 0b
S	Cham1	7 ± 0b	7.3 ± 0.6b	5.7 ± 2.3b	1 ± 0b	1 ± 0b	1 ± 0b
S	JK	14 ± 0a	16.7 ± 3.5a	23 ± 1a	3.3 ± 0.6a	4 ± 0a	3.7 ± 0.6a
S	Sebatel	7.3 ± 0.6b	8.5 ± 0.7b	6.7 ± 0.6b	1 ± 0b	1.5 ± 0.7b	1 ± 0b
	Genotype (G)	***	***	***	***	***	***
	Treatments (T)	***	***	***	ns	***	***
	G × T	*	***	***	**	***	***

Values are means ± standard deviations (n = 3). Means with the same letter in each column, within treatment, are not significantly different between genotypes (p < 0.05) (LSD test).

ns, not significant; LSD, least significant difference.

*, **, and *** indicate significance at p < 0.05, p < 0.01, and p < 0.001 levels, respectively.

The third stress event had a negative impact on NL of all genotypes for both treatments. After the fourth stress event, a major effect of salt stress was detected on NL rather than drought stress. It was recorded for all genotypes except for Azeghar, which had no significant difference between NL under control and drought conditions. Although abiotic stress decreased the number of leaves, J. Khetifa was characterized by many leaves during all experiments and under all conditions: control, drought, and salt.

After the second stress event, the number of tillers (NT) of all genotypes was not significantly affected by drought or salt conditions, except Cham1, which decreased NT under both stresses (**Table 2**). After the third stress event, the NT of J. Khetifa and Azeghar was reduced equally by drought and salt stresses.

However, in Cham1 and Sebatel, lower values of NT under salt stress than under drought were recorded. After the fourth stress event, salt had a more negative effect on NT than drought. However, the reduction of NT varied among genotypes. J. Khetifa had the greatest NT loss, but its number of tillers during the whole experiment under all conditions was significantly higher than that of the rest of the genotypes. Moreover, the number of tillers of Azeghar and Cham1 after the fourth drought stress was not significantly different from that under the control conditions.

### 3.2 Effects of cyclic drought and salt stresses on root traits

The total RL was significantly affected by treatments. Nevertheless, no significant difference among genotypes was detected after the first stress treatment (**Table 3**). At the early seedling stage, Cham1 and J. Khetifa significantly increased the RL under drought, compared with control and salt stress conditions. After the second stress event, Cham1 and J. Khetifa increased RL under drought and salt stresses, while Sebatel and Azeghar had no significant differences between control and stress conditions. After the third stress event, Azeghar decreased RL under drought and salt stresses, while Cham1 and J. Khetifa significantly decreased RL only under salt conditions. Sebatel continued to have no significant differences between control and stress conditions. Also, a statistically significant interaction after the fourth stress event in terms of the RL was found (**Table 3**). The RL of Azeghar was significantly negatively affected only by salt stress. While Cham1 decreased RL under both types of stress, under salt stress, the effect was statistically significantly higher. J. Khetifa showed the ability to grow roots after repeated stress treatment and had no differences between RL under stress and control conditions. The fourth drought and salt stresses negatively affected the RL of Sebatel at the same level.

**Table 3:** Total root length (RL) (cm) and root volume (RV) (cm^3^) after repeated drought and salt stresses (C, control; D, drought; S, salt).

T	Genotype	Total root length				Root volume			
		1st	2nd	3rd	4th	1st	2nd	3rd	4th
C	Azeghar	286 ± 58a	685 ± 107a	989 ± 84a	1,298 ± 309a	0.74 ± 0.19a	0.8 ± 0.28a	5.31 ± 1.14b	5.44 ± 0.72b
C	Cham1	285 ± 86a	333 ± 73c	1,056 ± 182a	1,457 ± 29a	0.82 ± 0.28a	0.57 ± 0.15a	7.01 ± 0.26b	5.24 ± 0.8b
C	JK	360 ± 9a	475 ± 105bc	973 ± 124a	756 ± 161b	0.47 ± 0.09a	0.57 ± 0.04a	9.73 ± 1.92a	21.61 ± 6.33a
C	Sebatel	382 ± 32a	578 ± 55ab	921 ± 231a	1,353 ± 299a	0.65 ± 0.14a	0.68 ± 0.18a	6.05 ± 1.6b	5.21 ± 0.09b
D	Azeghar	408 ± 145a	898 ± 175a	569 ± 186b	895 ± 94ab	0.57 ± 0.13a	0.63 ± 0.06b	0.97 ± 0.31b	1.39 ± 0.38b
D	Cham1	547 ± 22a	784 ± 93a	816 ± 125ab	806 ± 174b	0.67 ± 0.09a	0.86 ± 0.13ab	1.49 ± 0.47b	1.77 ± 0.29b
D	JK	526 ± 84a	948 ± 143a	887 ± 107a	1,141 ± 223a	0.74 ± 0.16a	1.03 ± 0.09a	2.18 ± 0.21a	4.51 ± 0.26a
D	Sebatel	402 ± 7a	820 ± 243a	806 ± 168ab	746 ± 111b	0.83 ± 0.07a	0.61 ± 0.24b	1.53 ± 0.07b	1.71 ± 0.54b
S	Azeghar	330 ± 44a	826 ± 52a	406 ± 238b	728 ± 150ab	0.49 ± 0.16a	1.28 ± 0.24a	0.59 ± 0.37b	0.55 ± 0.11b
S	Cham1	268 ± 41a	603 ± 23a	391 ± 29b	461 ± 235b	0.45 ± 0.12a	0.57 ± 0.02b	0.56 ± 0.06b	0.46 ± 0.2b
S	JK	324 ± 41a	890 ± 192a	641 ± 100b	875 ± 209a	0.55 ± 0.04a	1.07 ± 0.33ab	1.2 ± 0.35ab	1.24 ± 0.31a
S	Sebatel	348 ± 53a	737 ± 200a	1,051 ± 104a	828 ± 13a	0.55 ± 0.05a	0.98 ± 0.15ab	1.85 ± 1.02a	0.67 ± 0.14b
	Genotype (G)	ns	ns	*	ns	ns	ns	***	***
	Treatments (T)	***	***	***	***	*	**	***	***
	G × T	ns	ns	**	***	ns	**	**	***

Values are means ± standard deviations (n = 3). Means with the same letter in each column, within treatment, are not significantly different between genotypes (p < 0.05) (LSD test).

ns, not significant; LSD, least significant difference.

*, **, and *** indicate significance at p < 0.05, p < 0.01, and p < 0.001 levels, respectively.

The RV had no significant differences between control and stress conditions for all genotypes after the first stress event, except J. Khetifa, which significantly increased RV under drought stress (**Table 3**). After the second stress event, genotypes respond in terms of RV depending on the type of stress. This was highlighted by a significant G × T interaction. Azeghar increased RV under salt while Cham1 under drought. J. Khetifa significantly increased RV under both stresses, and Sebatel had no significant difference between control and stress conditions. The root volume was mostly affected negatively after the third stress event, even if the genotype response varied among drought and salt conditions. The RV of J. Khetifa, Azeghar, and Sebatel was similarly affected by both drought and salt stresses. Cham1 was more affected by salt than drought. After the fourth stress event, the RV of J. Khetifa and Azeghar was affected similarly by both drought and salt stresses, while that of Cham1 and Sebatel was more affected by salt than drought.

The RA (degree) varied significantly among genotypes in response to stress and over time (**Table 4**).

**Table 4:** The root angle (RA) (°) and the root surface area (SA) (cm^2^) after repeated drought and salt stresses (C, control; D, drought; S, salt).

T	Genotype	Root angle				Total root surface area			
		1st	2nd	3rd	4th	1st	2nd	3rd	4th
C	Azeghar	75.4 ± 1.2b	110.1 ± 4.4a	124.8 ± 3.5a	118.4 ± 3.7a	51.7 ± 11.9a	81.4 ± 7.8a	256 ± 33a	297 ± 53b
C	Cham1	107.2 ± 8.3a	105.9 ± 5.9a	114.9 ± 8.1a	120.5 ± 9.2a	54 ± 17.4a	48.5 ± 11.8c	304 ± 28a	309 ± 23b
C	JK	82.3 ± 3.8b	90.1 ± 3.3b	101.8 ± 1.6b	93.3 ± 5.1b	45.8 ± 4.4a	57.7 ± 4.2bc	345 ± 56a	444 ± 37a
C	Sebatel	109.1 ± 12.1a	106.1 ± 2.3a	113.9 ± 8.7a	117.2 ± 1.8a	55.6 ± 8.4a	69.9 ± 11.6ab	264 ± 68a	296 ± 32b
D	Azeghar	102 ± 8.6ab	121.2 ± 9.1ab	108.4 ± 5.3b	111.7 ± 3.7b	53.7 ± 15.7a	83.7 ± 8ab	83 ± 26b	124 ± 23b
D	Cham1	85.5 ± 9.3bc	115.5 ± 9b	121.1 ± 0.6a	125.4 ± 2.7a	68 ± 3.6a	92.2 ± 12ab	122 ± 24a	133 ± 13b
D	JK	88 ± 2.9c	119.9 ± 4.2ab	105.4 ± 4.1b	110.6 ± 2.7b	69.5 ± 9.3a	110.5 ± 5.6a	156 ± 15a	254 ± 32a
D	Sebatel	111.7 ± 7.4a	132.1 ± 4.9a	121.1 ± 1.4a	109.6 ± 6.7b	64.8 ± 2.2a	79.1 ± 27b	124 ± 14a	125 ± 19b
S	Azeghar	99.2 ± 3.4a	117 ± 7.9a	127.7 ± 1.4a	125.4 ± 6.8a	45 ± 9a	115 ± 14.1a	55 ± 33b	71 ± 12b
S	Cham1	93 ± 3.3a	102.9 ± 1.9b	120.9 ± 3.9ab	122.6 ± 5.3a	39.1 ± 8.3a	65.5 ± 0b	52 ± 5b	52 ± 24b
S	JK	67.2 ± 1.8b	95.4 ± 4.3b	104 ± 3.1c	118.8 ± 3.5a	47.1 ± 1.2a	109.1 ± 28.5a	98 ± 22ab	116 ± 26a
S	Sebatel	90.6 ± 7.6a	115.3 ± 2.7a	116.1 ± 11.6b	121.9 ± 6.3a	48.8 ± 6.1a	95.3 ± 20.3ab	154 ± 52a	83 ± 9ab
	Genotype (G)	***	***	***	***	ns	*	**	***
	Treatments (T)	*	***	***	***	***	***	***	***
	G × T	***	*	**	***	ns	*	ns	*

Values are means ± standard deviations (n = 3). Means with the same letter in each column, within treatment, are not significantly different between genotypes (p < 0.05) (LSD test).

ns, not significant; LSD, least significant difference.

*, **, and *** indicate significance at p < 0.05, p < 0.01, and p < 0.001 levels, respectively.

After the first stress event, the RA of Azeghar significantly widened under drought and salt conditions. The RA of Cham1 narrowed more under drought, and the RA of J. Khetifa narrowed more under salt conditions. The RA of Sebatel had no significant difference between control and stress conditions. After the second treatment, the RA of Azeghar and Cham1 was not significantly affected by drought and salt stresses compared with control conditions. The RA of J. Khetifa significantly widened under drought stress. Sebatel showed a wider RA under both stresses compared with the control, but the RA detected under drought was significantly wider than that under salt stress. After the third treatment, the RA of J. Khetifa, Sebatel, and Cham1 under both stresses had no significant differences compared with the control. Azeghar had a significantly narrower RA under drought compared to salt and control conditions. After the fourth treatment, the RA of Cham1 and Sebatel had no differences between control and stress conditions. Azeghar had a significantly narrower RA under drought stress. The RA of J. Khetifa under both stresses widened, but it was significantly wider under salt stress than under drought.

Azeghar and Sebatel root SA was not significantly affected after the first drought and salt stresses. The SA of J. Khetifa increased significantly only under drought conditions, and the SA of Cham1 significantly decreased only under salt conditions (**Table 4**). After the second treatment, Azeghar increased SA under salt stress, and Cham1 significantly increased SA under drought stress. The SA of J. Khetifa significantly increased under drought and salt treatments at the same level, and the SA of Sebatel had no significant differences between treatments and control. After the third stress event, J. Khetifa and Azeghar had a significant negative effect under drought and salt conditions on the SA. The SA of Cham1 also significantly decreased under both treatments, but salt applications had a stronger effect than drought. The SA of Sebatel significantly decreased only under drought conditions. After the fourth stress event, all genotypes had a significant negative impact of drought and salt on the SA. However, the salt treatment decreased the SA of Cham1 and J. Khetifa much more than drought.

The root average diameter (AG) varied significantly among genotypes in response to stress and over time (**Table 5**). The first stress event significantly decreased the AG for Azeghar and Cham1 under both stresses. The AG of Cham1 had a stronger negative response to drought stress than to salt stress. The AG of J. Khetifa and Sebatel was not significantly affected after the first and second stress treatments, while Cham1 AG significantly decreased under both stresses. The AG of Azeghar significantly decreased only under drought conditions. After the third stress event, the AG of Azeghar, Sebatel, and Cham1 significantly decreased under both stresses equally, while the AG of J. Khetifa was more affected by salt stress. After the fourth stress event, salt negatively affected the AG of all genotypes more than drought stress, except the AG of J. Khetifa, which was affected by both types of stress equally. The number of root tips (TI) varied significantly in response to drought and salt stresses among genotypes and over time (**Table 5**). Though J. Khetifa had the lowest TI under control conditions, after the first stress event, the TI significantly increased only under drought conditions. However, the TI of Cham1 and Sebatel decreased significantly only under salt stress. The TI of Azeghar had no significant differences between control and stress conditions. After the second stress event, the response to different stress varied among all genotypes. The TI of J. Khetifa and Cham1 increased significantly under drought and salt stresses, but the TI of Cham1 increased more under drought compared with salt. The TI of Azeghar increased significantly only under drought conditions, and the TI of Sebatel had no significant differences between control and stress conditions. After the third stress event, the TI of J. Khetifa and Sebatel had no significant differences between control and stress treatments. However, the TI of Azeghar and Cham1 decreased significantly only under salt stress. After the fourth stress event, the TI of Azeghar had no significant differences between control and stress conditions. The TI of Cham1 decreased similarly under both stresses, and Sebatel decreased significantly only under drought. The TI of J. Khetifa significantly increased only under salt treatment.

**Table 5:** The root average diameter (mm) and the number of root tips (TI) after repeated drought and salt stresses (C, control; D, drought; S, salt).

T	Genotype	Root average diameter				Number of root tips			
		1st	2nd	3rd	4th	1st	2nd	3rd	4th
C	Azeghar	0.57 ± 0.03a	0.39 ± 0.1a	0.82 ± 0.08b	0.74 ± 0.07b	455 ± 27c	1,243 ± 287a	1,989 ± 217a	2,723 ± 850ab
C	Cham1	0.6 ± 0.02a	0.46 ± 0.01a	0.93 ± 0.08b	0.67 ± 0.06b	896 ± 110a	633 ± 94a	2,009 ± 312a	3,614 ± 423a
C	JK	0.41 ± 0.05b	0.39 ± 0.06a	1.13 ± 0.04a	1.93 ± 0.45a	538 ± 42c	1,188 ± 406a	2,218 ± 309a	1,726 ± 194b
C	Sebatel	0.46 ± 0.03b	0.38 ± 0.05a	0.91 ± 0.01b	0.71 ± 0.09b	733 ± 37b	1,326 ± 507a	1,704 ± 385a	2,870 ± 730a
D	Azeghar	0.43 ± 0.04b	0.3 ± 0.04b	0.47 ± 0.05a	0.44 ± 0.04b	666 ± 231b	2,493 ± 913a	1,314 ± 329b	2,044 ± 106a
D	Cham1	0.4 ± 0.03b	0.37 ± 0.01a	0.48 ± 0.08a	0.53 ± 0.08b	802 ± 39ab	2,195 ± 210a	2,299 ± 524a	1,915 ± 411a
D	JK	0.42 ± 0.06b	0.37 ± 0.04a	0.56 ± 0.03a	0.71 ± 0.05a	973 ± 78a	2,897 ± 885a	1,970 ± 168ab	2,359 ± 509a
D	Sebatel	0.52 ± 0.02a	0.3 ± 0.02b	0.5 ± 0.05a	0.54 ± 0.1b	819 ± 6ab	2,116 ± 430a	1,799 ± 489ab	1,714 ± 560a
S	Azeghar	0.43 ± 0.06a	0.44 ± 0.03a	0.43 ± 0.02a	0.31 ± 0.03b	473 ± 26ab	1,510 ± 119b	921 ± 558b	1,909 ± 606bc
S	Cham1	0.46 ± 0.03a	0.35 ± 0.01b	0.43 ± 0.02a	0.37 ± 0.03ab	367 ± 74b	1,362 ± 21b	866 ± 97b	1,139 ± 592c
S	JK	0.46 ± 0.05a	0.39 ± 0.03ab	0.48 ± 0.04a	0.42 ± 0.04a	515 ± 10a	2,399 ± 143a	1,805 ± 316a	3,073 ± 541a
S	Sebatel	0.45 ± 0.01a	0.42 ± 0.03a	0.46 ± 0.11a	0.32 ± 0.03b	592 ± 97a	1,480 ± 352b	2,492 ± 13a	2,580 ± 190ab
	Genotype (G)	*	ns	***	***	**	ns	**	ns
	Treatments (T)	***	**	***	***	***	***	**	**
	G × T	***	*	*	***	***	ns	***	***

Values are means ± standard deviations (n = 3). Means with the same letter in each column, within treatment, are not significantly different between genotypes (p < 0.05) (LSD test).

ns, not significant; LSD, least significant difference.

*, **, and *** indicate significance at p < 0.05, p < 0.01, and p < 0.001 levels, respectively.

After the first stress event, the number of root FR of J. Khetifa and Cham1 increased significantly only under drought conditions, while Azeghar and Sebatel did not exhibit significant differences between the treatments (**Table 6**). After the second stress event, the FR of J. Khetifa and Azeghar increased similarly under both drought and salt, while Cham1 significantly increased FR only under drought conditions. The FR of Sebatel had no significant differences between control and stress conditions. After the third stress event, drought and salt stress events significantly reduced the FR of Azeghar, Cham1, and J. Khetifa, but at different levels. The FR of Azeghar decreased similarly under both stresses, while the FR of Cham1 and J. Khetifa decreased significantly more under salt than drought. After the third stress event, the FR of Sebatel still had no significant differences between the control and both stress conditions. After the fourth stress event, the FR of Azeghar, Cham1, and Sebatel decreased under the two treatments, while the FR of J. Khetifa had no significant differences between control and stress conditions.

**Table 6:** The number of root forks (FR) and the number of root crossings (CR) after repeated drought and salt stresses (C, control; D, drought; S, salt).

T	Genotype	Number of root forks				Number of root crossings			
		1st	2nd	3rd	4th	1st	2nd	3rd	4th
C	Azeghar	2,318 ± 472a	4,990 ± 914a	12,865 ± 2,083a	18,389 ± 5,170ab	225 ± 54b	956 ± 487a	1,313 ± 159a	2,579 ± 846a
C	Cham1	2,090 ± 1,290a	2,774 ± 542b	13,726 ± 1,967a	21,535 ± 1,692a	188 ± 168b	421 ± 162a	1,349 ± 265a	3,634 ± 649a
C	JK	3,027 ± 508a	4,651 ± 511a	15,185 ± 3,056a	12,982 ± 2,057b	511 ± 165a	942 ± 204a	1,355 ± 318a	1,081 ± 381b
C	Sebatel	2,924 ± 370a	4,587 ± 897a	11,618 ± 3,490a	19,550 ± 4,570ab	401 ± 52ab	857 ± 136a	1,033 ± 381a	2,782 ± 962a
D	Azeghar	3,026 ± 795b	7,069 ± 955b	5,757 ± 2,285b	9,494 ± 2,480b	465 ± 175b	1,854 ± 470ab	969 ± 459a	1,760 ± 554a
D	Cham1	4,005 ± 567ab	6,204 ± 562b	8,520 ± 1,651ab	10,006 ± 2,240b	720 ± 178ab	1,111 ± 121b	1,451 ± 264a	1,832 ± 610a
D	JK	4,677 ± 602a	9,924 ± 1,236a	10,519 ± 1,930a	15,941 ± 3,782a	772 ± 154a	2,052 ± 564a	1,579 ± 353a	2,195 ± 669a
D	Sebatel	3,350 ± 351ab	6,256 ± 2,456b	8,299 ± 1,758ab	9,274 ± 1,659b	455 ± 62b	1,361 ± 639ab	1,328 ± 500a	1,590 ± 437a
S	Azeghar	2,274 ± 468ab	6,606 ± 143ab	3,332 ± 2,065c	5,410 ± 1,180b	300 ± 79ab	1,056 ± 97ab	511 ± 338c	1,257 ± 330ab
S	Cham1	1,627 ± 200b	3,547 ± 242b	3,457 ± 598c	3,834 ± 2,005b	189 ± 11b	694 ± 26b	562 ± 152c	828 ± 416b
S	JK	2,706 ± 252a	8,180 ± 1,987a	6,432 ± 1,397b	9,741 ± 3,287a	340 ± 45a	1,543 ± 423a	1,007 ± 158b	1,970 ± 770a
S	Sebatel	2,597 ± 551a	6,100 ± 2,120ab	10,827 ± 1,684a	6,623 ± 1,228ab	331 ± 93a	1,122 ± 503ab	1,934 ± 206a	1,505 ± 200ab
	Genotype (G)	**	**	*	ns	**	*	*	ns
	Treatments (T)	***	***	***	***	***	***	*	***
	G × T	ns	ns	*	**	*	ns	**	**

Values are means ± standard deviations (n = 3). Means with the same letter in each column, within treatment, are not significantly different between genotypes (p < 0.05) (LSD test).

ns, not significant; LSD, least significant difference.

*, **, and *** indicate significance at p < 0.05, p < 0.01, and p < 0.001 levels, respectively.

After the first stress event, the number of root CR of J. Khetifa, Azeghar, and Cham1 significantly increased only under drought, while the number of root CR of Sebatel had no significant differences between control and stress conditions (**Table 6**). After the second stress event, the CR of Khetifa and Azeghar significantly increased only under drought, while the CR of Cham1 significantly increased under drought and salt stresses. The CR of Sebatel still had no significant differences between the control and both stresses. After the third stress event, the CR of Azeghar and Cham1 significantly decreased only under salt stress, while the CR of J. Khetifa and Sebatel had no significant differences between control and stress conditions. After the fourth stress event, the CR of Cham1 and Sebatel significantly decreased under drought and salt, while the CR of Azeghar significantly decreased only under salt conditions. The CR of J. Khetifa had no significant differences among the treatments.

The RDW significantly decreased under drought and salt for all genotypes. Despite significant losses in RDW among all genotypes, at the end of the experiment, J. Khetifa had significantly bigger RDW under control, drought, and salt conditions (**Table 7**).

**Table 7:** The root dry weight (RDW) (g) and the root/shoot ratio (RS) after repeated drought and salt stresses (C, control; D, drought; S, salt).

T	Genotype	Root dry weight		Root/shoot ratio	
		3rd	4th	3rd	4th
C	Azeghar	0.53 ± 0.12b	0.82 ± 0.08b	0.25 ± 0.06a	0.11 ± 0.01b
C	Cham1	0.83 ± 0.11a	1.09 ± 0.14b	0.42 ± 0.16a	0.15 ± 0.02b
C	JK	0.86 ± 0.2a	2.1 ± 0.22a	0.33 ± 0.08a	0.28 ± 0.05a
C	Sebatel	0.62 ± 0.15ab	1.09 ± 0.28b	0.3 ± 0.02a	0.15 ± 0.04b
D	Azeghar	0.17 ± 0.06b	0.29 ± 0.11b	0.33 ± 0.05b	0.16 ± 0.02b
D	Cham1	0.26 ± 0.06ab	0.42 ± 0.04b	0.51 ± 0.04a	0.21 ± 0.03b
D	JK	0.3 ± 0.07a	0.98 ± 0.07a	0.48 ± 0.06a	0.42 ± 0.05a
D	Sebatel	0.25 ± 0.04ab	0.34 ± 0.1b	0.38 ± 0.03b	0.16 ± 0.02b
S	Azeghar	0.1 ± 0.06b	0.15 ± 0.02b	0.11 ± 0.04a	0.07 ± 0.01d
S	Cham1	0.11 ± 0.02b	0.12 ± 0.06b	0.15 ± 0.04a	0.12 ± 0.01b
S	JK	0.21 ± 0.06ab	0.32 ± 0.05a	0.2 ± 0.03a	0.21 ± 0.01a
S	Sebatel	0.31 ± 0.11a	0.19 ± 0.04b	0.2 ± 0.09a	0.09 ± 0c
	Genotype (G)	**	***	**	***
	Treatments (T)	***	***	***	***
	G × T	*	***	ns	**

Values are means ± standard deviations (n = 3). Means with the same letter in each column, within treatment, are not significantly different between genotypes (p < 0.05) (LSD test).

ns, not significant; LSD, least significant difference.

*, **, and *** indicate significance at p < 0.05, p < 0.01, and p < 0.001 levels, respectively.

After the third stress event, the root/shoot ratio (RS) of Azeghar and Cham1 significantly decreased only under salt stress. The RS of J. Khetifa also significantly decreased under salt stress but significantly increased under drought (**Table 7**). The RS of Sebatel had no significant differences between control and stress conditions. After the fourth stress event, the RS of Azeghar significantly decreased under salt stress but significantly increased under drought. The RS of J. Khetifa and Cham1 significantly increased only under drought stress, while the RS of Sebatel significantly decreased only under salt stress.

### 3.3 Genotype response to drought and salt during the experiment

At the early seedling stage after the first drought and salt stress application, root and shoot traits of Sebatel had no significant differences between control and stress conditions, except for the TI, which decreased under salt stress (**Table 8**). In turn, J. Khetifa significantly increased some traits, including RL, RV, SA, TI, FR, and CR, but only under drought conditions.

**Table 8:** Genotypic response after the first drought and salt application.

Genotype	PH	RV	AG	TI	CR	FR	RL	SA	RA
Azeghar	no	no	∨DS	no	∧D	no	no	no	∧DS
Cham1	∨S	no	∨DS	∨S	∧D	∧D	∧D	∨S	∨D
J. Khetifa	no	∧D	no	∧D	∧D	∧D	∧D	∧D	∨S
Sebatel	no	no	no	∨S	no	no	no	no	no

(no), no significant differences between stress and control conditions; (∧), significantly increased; (∨), significantly decreased; (D), under drought; (S), under salt; (DS), under drought and salt; PH, plant height; RV, root volume; AG, root average diameter; TI, number of tips; CR, number of crossings; FR, number of forks; RL, total root length; SA, root surface area; RA, root angle.

Moreover, at the early seedling stage, J. Khetifa had a significantly narrower root angle under salt stress. Azeghar, after the first (drought and salt) stress application, had widened RA and decreased AG under both conditions and increased CR, but only under drought.

Cham1 seedlings had both positive and negative effects on most of the traits. Salt stress negatively affected PH, SA, and TI. Under drought conditions, the RA of Cham1 significantly narrowed, and parameters such as RL, FR, and CR increased. The AG of Cham1 was reduced by both stresses, but significantly more under drought. After the second stress event, Sebatel had no significant differences between control and stress or all traits except the RA, which widened under drought and salt conditions. Some traits of J. Khetifa significantly increased under both types of stress, such as RL, RV, SA, NL, TI, and FR (**Table 9**). However, the RA and CR increased only under drought stress. After the second stress event, only the PH of J. Khetifa was affected negatively by drought and salt stresses. Azeghar’s response to the different types of stress was very variable. Traits such as PH, RV, and SA significantly increased only under salt stress, while TI and CR significantly increased only under drought conditions.

**Table 9:** Genotypic response after the second drought and salt application.

Genotype	NL	NT	PH	RV	AG	TI	CR	FR	RL	SA	RA
Azeghar	∧DS	no	∧S	∧S	∨D	∧D	∧D	∧DS	no	∧S	no
Cham1	no	∨DS	∨DS	∧D	∨DS	∧DS	∧DS	∧D	∧DS	∧D	no
J. Khetifa	∧DS	no	∨DS	∧DS	no	∧DS	∧D	∧DS	∧DS	∧DS	∧D
Sebatel	no	no	no	no	no	no	no	no	no	no	∧**D**S

(no), no significant differences between stress and control conditions; (∧), significantly increased; (∨), significantly decreased; (D), under drought; (S), under salt; (DS), under drought and salt; NL, number of leaves; NT, number of tillers; PH, plant height; RV, root volume; AG, root average diameter; TI, number of tips; CR, number of crossings; FR, number of forks; RL, total root length; SA, root surface area; RA, root angle.

The NL and FR significantly increased under both stresses, while AG under drought significantly decreased. After the second stress event, Cham1 had the largest number of negatively affected traits compared to other genotypes, including PH, NT, and AG. However, under both types of stresses, Cham1 had significantly increased RL, TI, and CR and, only under drought, significantly increased RV, SA, and FR.

After the third stress event, Sebatel had no significant differences between control and stress conditions for half of the evaluated traits, except for NL, NT, RV, and AG, which significantly decreased under drought and salt (**Table 10**).

**Table 10:** Genotypic response after the third drought and salt application.

Genotype	NL	NT	PH	RV	AG	TI	CR	FR	RL	SA	RA	RDW	RS
Azeghar	∨DS	∨DS	∨D	∨DS	∨DS	∨S	∨S	∨DS	∨DS	∨DS	∨D	∨DS	∨S
Cham1	∨DS	∨DS	∨DS	∨D**S**	∨DS	∨S	∨S	∨DS	∨S	∨D**S**	no	∨DS	∨S
J. Khetifa	∨DS	∨DS	∨S	∨DS	∨D**S**	no	no	∨DS	∨S	∨DS	no	∨DS	∧D∨S
Sebatel	∨DS	∨DS	no	∨DS	∨DS	no	no	no	no	∨D	no	∨DS	no

(no), no significant differences between stress and control conditions; (∧), significantly increased; (∨), significantly decreased; (D), under drought; (S), under salt; (DS), under drought and salt; NL, number of leaves; NT, number of tillers; PH, plant height; RV, root volume; AG, root average diameter; TI, number of tips; CR, number of crossings; FR, number of forks; RL, total root length; SA, root surface area; RA, root angle; RDW, root dry weight; RS, root/shoot ratio.

However, the SA and RDW of Sebatel significantly decreased only under drought conditions. After the third stress event, J. Khetifa had no significant differences between control and stress for traits such as TI, CR, and RA. The PH and RL of J. Khetifa were more sensitive to salt than to drought. Interestingly, the RS of J. Khetifa significantly increased under drought stress but significantly decreased under salt stress. After the third stress event, Cham1 had no significant differences between control and stress for the RA. All the other traits significantly decreased after both types of stresses, except TI, CR, and RS, which were more sensitive to salt than drought. All traits of Azeghar were significantly affected but varied regarding types of stress. The PH and RA of Azeghar were more sensitive to drought than salt, and TI, CR, and RS were more sensitive to salt than drought.

After the fourth stress event, all traits in all genotypes were significantly affected by both or just one of the stresses. However, J. Khetifa had no significant differences between control and stress conditions for traits such as RL, CR, and FR (**Table 11**). Moreover, under salt, J. Khetifa had significantly increased TI. After the fourth stress event, RA and RS varied the most between genotypes in response to stress. The root angle of Cham1 and Sebatel had no statistical differences between control and stress conditions.

**Table 11:** Genotypic response after the fourth drought and salt application.

Genotype	NL	NT	PH	RV	AG	TI	CR	FR	RL	SA	RA	RDW	RS
Azeghar	∨S	∨S	∨DS	∨DS	∨DS	no	∨S	∨DS	∨S	∨DS	∨D	∨DS	∧D∨S
Cham1	∨DS	∨DS	∨DS	∨DS	∨DS	∨DS	∨DS	∨DS	∨DS	∨DS	no	∨DS	∧D
J. Khetifa	∨DS	∨DS	∨D	∨DS	∨DS	∧S	no	no	no	∨DS	∧DS	∨DS	∧D
Sebatel	∨DS	∨DS	∨DS	∨DS	∨DS	∨D	∨DS	∨DS	∨DS	∨DS	no	∨DS	∨S

(no), no significant differences between stress and control conditions; (∧), significantly increased; (∨), significantly decreased; (D), under drought; (S), under salt; (DS), under drought and salt; NL, number of leaves; NT, number of tillers; PH, plant height; RV, root volume; AG, root average diameter; TI, number of tips; CR, number of crossings; FR, number of forks; RL, total root length; SA, root surface area; RA, root angle; RDW, root dry weight; RS, root/shoot ratio.

However, the RA of J. Khetifa widened significantly under both stresses, and the RA of Azeghar narrowed only under drought. The RS of Cham1 and J. Khetifa significantly increased under drought, and the RS of Sebatel decreased significantly only under salt. Interestingly, the RS of Azeghar significantly increased under drought and significantly decreased under salt stress.

### 3.4 SSR marker analysis

The genetic analysis of the 11 SSR markers showed a total of 38 alleles across the four genotypes; four SSRs (i.e., gwm234, wmc727, gwm459, and gwm499) identified two loci, which were named “a” and “b”. The number of alleles per locus ranged from one for wmc727b, gwm459b, and gwm499a to five for cfa2257 (**Table 12**). The expected heterozygosis (He), excluding the three markers with one allele per locus, which was 0, ranged from 0.38 for gwm234a and gwm636 to 0.75 for cfa2086, cfa2257, and gwm427. PIC was observed to differ significantly, ranging from 0.43 (gwm234a and gwm636) to 0.86 (cfa2086, cfa2257, and gwm427), revealing that these markers have the required properties to be used in diversity studies for root traits. The high Shannon Information Index (I) detected values ranging from 0.56 to 1.49, reflecting the heterozygous nature of the genotypes studied for these markers. The percentage of polymorphic bands was 20% for J. Khetifa and Cham1 and 27% for Azeghar and Sebatel (data not shown).

**Table 12:** Genetic diversity based on the 11 SSR markers with 15 different loci.

Locus	Na	I	Ho	He	PIC	Size, bp
cfa2086	4	1.39	0	0.75	0.86	223–277
wms5	3	1.04	0	0.63	0.71	180–190
gwm234a	2	0.56	0	0.38	0.43	214–218
gwm234b	2	0.69	0	0.50	0.57	242–256
wmc727a	2	0.69	1	0.50	0.57	88–96
wmc727b	1	0.00	0	0.00	0.00	231
cfa2257	5	1.49	1	0.75	0.86	104–156
wms205	2	0.69	1	0.50	0.57	157–170
gwm427	4	1.39	0	0.75	0.86	201–256
gwm573.2	4	1.26	1	0.69	0.79	181–240
gwm636	2	0.56	0	0.38	0.43	110–125
gwm459a	2	0.69	0	0.50	0.57	137–153
gwm459b	1	0.00	0	0.00	0.00	292
gwm499a	1	0.00	0	0.00	0.00	91
gwm499b	3	1.04	0	0.63	0.71	147–197

Different loci of same marker are highlighted as “a” and “b”.

Na, no. of alleles; I, Shannon’s Information Index = −1 * Sum (pi * Ln (pi)); Ho, observed heterozygosity, He, expected heterozygosity; PIC, polymorphism information content; SSR, single sequence repeat.

Interestingly, all the genotypes had at least one private allele, and the three markers with the highest PIC registered (cfa2086, cfa2257, and gwm427) had also the highest number of private alleles. The number of private alleles detected was similar for all four genotypes, although the landrace J. Khetifa had the highest number of private alleles.

**Figure 3:**
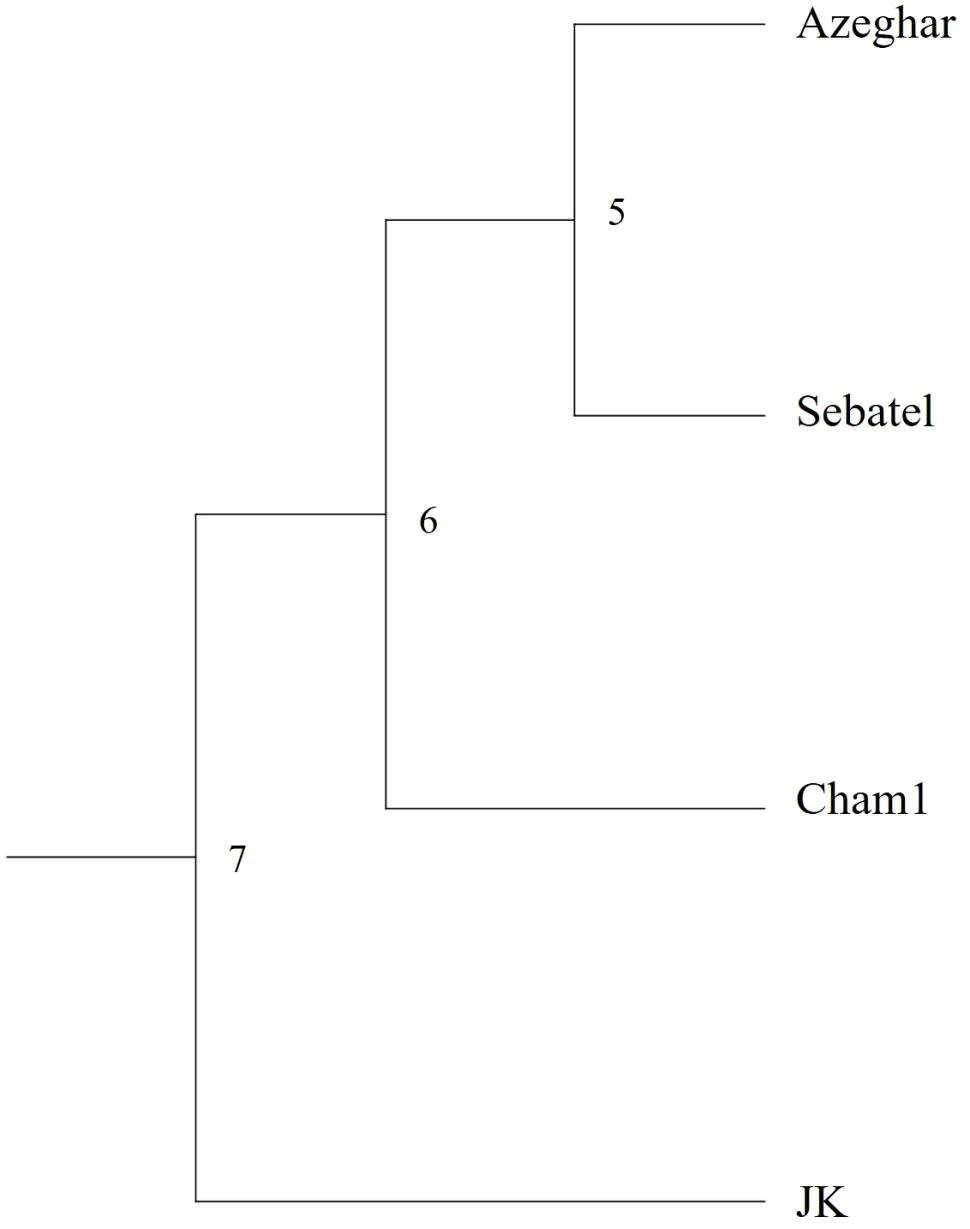
UPGMA phenogram of Nei’s genetic distance among the four screened genotypes based on the genetic analysis of 11 single sequence repeat (SSR) markers.

The cluster analysis of Nei’s genetic distance (Nei, 1972) showed that J. Khetifa (JK) was grouped alone, while the other three genotypes were grouped together (**Figure 3**). This marks the clear differences between landraces and modern varieties, which were also clear for the root angle trait, where J. Khetifa showed a significantly narrower root angle than other genotypes under all treatments and for each phenological stage.

## 4 Discussion

Durum wheat production in the Mediterranean environment depends upon rainfall during the growing season, as most precipitation falls in winter. The Mediterranean basin is characterized by high volatility in rainfall distribution; the fluctuation in rainfall during the growing season results in cyclic water availability. Moreover, due to climate changes in this region, the sea level rises, increasing salt wedge and increasing salt concentrations in farmlands near cost (Corwin, 2021). Salt stress represents a serious threat, as it has a negative impact on wheat plants’ growth and yield, affecting leaf area, root and shoot biomass, and grain number and size (Talaat and Hanafy, 2022). Root plasticity plays a crucial role in adaptation to abiotic stress, while root diversity among landraces and modern varieties can play a key role in durum wheat breeding programs. Since understanding the complex genetic mechanisms behind the stress-escape strategies is a major challenge in breeding programs for drought and salt tolerance, accurate phenotyping is the key to developing new useful material (Gilliham et al., 2017). Durum wheat landraces, such as J. Khetifa used in the present study, are an essential group of genetic resources for breeding due to their great genetic diversity, good adaptation to the local environment, and resilience to abiotic and biotic stresses (Nachit et al., 2001; Talas et al., 2011; Mondini et al., 2012; Mohammadi et al., 2015; Soriano et al., 2016). In addition, Roselló et al. (2019) observed a huge variability in the seminal root system among 160 durum wheat landraces from 21 Mediterranean countries. Boudiar et al. (2019) reported the superiority of early drought tolerance of Algerian durum wheat landraces, which are good resources for breeding. Moreover, in the study of van der Bom et al. (2023), the genotype with a narrow root angle exhibited enhanced deep soil exploration during early growth, showing a propensity for more rapid acquisition of phosphorus placed in a deep fertilizer layer. In our study, the landrace J. Khetifa after the first application of drought and salt was more sensitive to water deficit and improved important root parameters, such as total RL, RV, root SA, TI, FR, and CR. In comparison with other genotypes, J. Khetifa had the strongest positive response to stress regarding root traits, particularly at the early seedling stage. Meanwhile, the RL, CR, and FR of Cham1 increased; only the CR of Azeghar increased; Sebatel did not respond to any stress. J. Khetifa showed the same significant positive response for RL, RV, SA, TI, NL, and FR after the second stress application too, but under both stresses. After the third stress event, negative effects were observed for all genotypes, but the sensitivity of traits to individual stress differed. Although all genotypes responded differently to both stresses at different development stages during the whole experiment, J. Khetifa had significantly higher NL, NT, RL, RV, SA, AG, RDW, RS, and FR than the other genotypes at the end of the experiment. Therefore, it can be concluded that a positive response at the early seedling stage and strong early vigor of J. Khetifa helped in the adaptation to abiotic stresses in more advanced stages. The root system architecture and its plasticity under stressful environments are complex and controlled by many genes. In addition, root phenotyping is time-consuming and not easy to manage, particularly in advanced stages. Therefore, the identification of molecular markers related to QTLs involved in root morphology is useful for the early marker-assisted selection of genotypes suitable for environments at high risk of drought and salt stresses. In the present study, 11 root-related SSR markers were selected and used to characterize four durum wheat genotypes and to evidence the differences of one landrace with three modern varieties. The study previously conducted by Urbanavičiūtė et al. (2023) enhanced the high association of the SSR marker here used with known QTLs involved in root trait plasticity (**Supplementary Table S2**). The private alleles found in the landrace (**Table 13**) may represent a useful resource of molecular material for the selection or breeding for drought- and salt-affected environments. The selection for cfa2086, wms5 gwm234, gwm427, gwm573, and gwm499 can be useful to detect the introgression in new cultivars or progeny for the related QTLs for a better adaptation to drought and salt stresses. The marker cfa2086 and the marker gwm427 were found to be strictly associated with root growth angle (RGA) of QTLs, respectively, QRga.ubo-2A.3 and QRga.ubo-6A.2, which are associated with total root diameter and root angle traits and co-localized with QTLs related to grain yield and grain size (Maccaferri et al., 2016). The QTLs associated with wms5 are related to the root system architecture (RSA) for traits such as total root length and lateral root length, while the QTLs mtaq-5B.1 and mtaq-5B.2 were found associated with respectively total lateral root length and primary root volume trait for the markers gwm499 and gwm234 (Roselló et al., 2019). The marker gwm573.2 was found to be associated with the QTL MQTL7B.3 associated with root-related traits and yellow pigment content in terms of quality (Soriano et al., 2021). Moreover, as demonstrated in Urbanavičiūtė et al. (2023), the marker wms5 has given a consistent result, discriminating for the root angle the genotype J. Khetifa, which showed a narrower root angle with the private allele of 190 bp. A high positive correlation between the root angle of plants grown in pots under controlled environments and the root angle of plants, at the same phenological stage, grown in the field was already found in Urbanavičiūtė et al. (2022a). The markers in this study used for discriminating the root angle are a resource for the selection of genotypes with narrow root angles, hence with improved drought tolerance. Moreover, Thapa et al. (2017) found a significant negative effect of salinity on the root parameters of plants grown in the field. A major impact, growing with soil depth, was registered for traits such as root surface area, root length, root volume, and root biomass. In terms of marker-assisted selection, the alleles of the screened SSR markers could help in the selection of drought- and salt-tolerant genotypes. However, further studies are needed to state that these markers can be used to transfer stress tolerance alleles from tolerant genotypes to susceptible ones.

**Table 13:** Markers’ private alleles.

Locus	Allele	Frequency	Found in
cfa2086	223	1	Sebatel
cfa2086	269	1	Cham1
cfa2086	277	1	J. Khetifa
cfa2086	234	1	Azeghar
wms5	185	1	Cham1
wms5	190	1	J. Khetifa
gwm234	214	1	J. Khetifa
cfa2257	150	0.5	Sebatel
cfa2257	121	1	Cham1
cfa2257	142	0.5	Azeghar
cfa2257	104	0.5	Azeghar
gwm427	237	1	Sebatel
gwm427	214	1	Cham1
gwm427	256	1	J. Khetifa
gwm427	201	1	Azeghar
gwm573.2	236	0.5	J. Khetifa
gwm573.2	181	0.5	Azeghar
gwm637	125	1	Sebatel
gwm499	147	1	Sebatel
gwm499	154	1	J. Khetifa

## 5 Conclusion

The present study shows how a high capacity of plasticity, with the consequent ability to modify the root architecture from an early stage, is useful, for plants to better tolerate hydric stress; hence, this characteristic should be considered in breeding programs to develop varieties for drought and/or salty environments. Moreover, the landraces, such as J. Khetifa and its alleles, still represent an important resource for the introgression of desirable traits into modern cultivars. Due to the complexity of the genetic regulation of abiotic stress tolerance, marker-assisted selection (MAS) is a high-potential tool for the identification of QTLs related to the trait of interest. The combination of high-throughput phenotyping with SSR screening can provide important highlights to increase the efficiency of stress-related breeding programs.

## Data availability statement

The original contributions presented in the study are included in the article/**Supplementary Material**. Further inquiries can be directed to the corresponding author.

## Author contributions

LB: Data curation, Formal analysis, Investigation, Methodology, Software, Visualization, Writing – original draft, Writing – review & editing. IU: Data curation, Formal analysis, Investigation, Methodology, Software, Visualization, Writing – original draft, Writing – review & editing. MP: Conceptualization, Formal analysis, Funding acquisition, Methodology, Project administration, Supervision, Validation, Writing – review & editing.
